# The use of geographic information system and 1860s cadastral data to model agricultural suitability before heavy mechanization. A case study from Malta

**DOI:** 10.1371/journal.pone.0192039

**Published:** 2018-02-07

**Authors:** Gianmarco Alberti, Reuben Grima, Nicholas C. Vella

**Affiliations:** 1 Department of Classics and Archaeology, University of Malta, Msida, Malta; 2 Department of Conservation and Built Heritage, University of Malta, Msida, Malta; University of Porto, PORTUGAL

## Abstract

The present study seeks to understand the determinants of land agricultural suitability in Malta before heavy mechanization. A GIS-based Logistic Regression model is built on the basis of the data from mid-1800s cadastral maps (cabreo). This is the first time that such data are being used for the purpose of building a predictive model. The maps record the agricultural quality of parcels (ranging from good to lowest), which is represented by different colours. The study treats the agricultural quality as a depended variable with two levels: optimal (corresponding to the good class) vs. non-optimal quality (mediocre, bad, low, and lowest classes). Seventeen predictors are isolated on the basis of literature review and data availability. Logistic Regression is used to isolate the predictors that can be considered determinants of the agricultural quality. Our model has an optimal discriminatory power (AUC: 0.92). The positive effect on land agricultural quality of the following predictors is considered and discussed: sine of the aspect (odds ratio 1.42), coast distance (2.46), Brown Rendzinas (2.31), Carbonate Raw (2.62) and Xerorendzinas (9.23) soils, distance to minor roads (4.88). Predictors resulting having a negative effect are: terrain elevation (0.96), slope (0.97), distance to the nearest geological fault lines (0.09), Terra Rossa soil (0.46), distance to secondary roads (0.19) and footpaths (0.41). The model isolates a host of topographic and cultural variables, the latter related to human mobility and landscape accessibility, which differentially contributed to the agricultural suitability, providing the bases for the creation of the fragmented and extremely variegated agricultural landscape that is the hallmark of the Maltese Islands. Our findings are also useful to suggest new questions that may be posed to the more meagre evidence from earlier periods.

## Introduction

The study of past landscapes and the way they have evolved over long periods of time is key to understanding the changing relationship between humans and their environment. Approaches to the study of long-term landscape change is increasingly making use of an ever-growing array of different tools and data-sets, ranging from environmental reconstruction to ethnographic comparison, from to surface survey to map regression. The present study was undertaken in the context of the five-year ERC-funded FRAGSUS project, which examines fragility and sustainability in small island contexts, with a focus of human-environment interactions in prehistoric Malta. The sheer density of human activity on Malta over the past 7,000 years makes the reconstruction of past environments extremely challenging, as the evidence is at best fragmentary, and often obliterated by subsequent erosion and anthropogenic activity [1–3]. The challenge increases exponentially for more remote periods in time.

While the primary focus of FRAGSUS is the prehistoric environment, a sound understanding of the subsequent evolution of the landscape was considered essential and useful for a number of key reasons: first, because they form part of the same landscape palimpsest that can only be understood in diachronic terms; second, to allow more informed predictions of where evidence of earlier landscapes may be preserved; third, because different cultural responses in better documented periods may suggest new questions that may be posed to the more meagre evidence from earlier periods, and enrich their interpretations.

Archival records preserved from the early modern period onwards include census records and cadastral maps that may contain detailed records of ownership, productivity and yield of land, range of crops and size of herds, as well as human demography. This record, which tends to be increasingly rich in coverage and detail in more recent centuries, offers ample opportunities for the historical reconstruction of early modern landscapes. In a study of another Mediterranean small island context [4], it has been noted how the uneven nature of such records may give rise to a form of analytical exceptionalism which privileges better-documented periods, and *that there is an obvious risk that the unusually detailed historical & ethnographic evidence available for the late eighteenth to twentieth centuries will lead to some gross methodological differences compared to earlier periods* that may *skew our practical interpretations*. These risks notwithstanding, the same researchers conclude that *the abundant historical records and standing remains of the late eighteenth to twentieth centuries are a resource that it would be foolish to ignore* [4].

Bearing the above pitfalls in mind, but also mindful of the opportunities presented by early modern archival records, the present study examined a comprehensive survey undertaken by the British colonial government in Malta in the 1860s to produce a highly accurate and detailed terrier, or cabreo. It consists of around 750 ink and watercolour drawings of all Crown property in Malta [5]. Bound in three large-format volumes with plans of government properties on the island of Malta, and a fourth with properties on the island of Gozo, these cadastral records, referred to below collectively as the cabreo, are today held in the National Archives of Malta in Rabat. The records of rural properties contained in the cabreo offer a rare and detailed glimpse into the organisation of the productive landscape in the early modern period. The detailed documentation of the productivity or land quality of different parcels of land, described in detail below, presents the researcher with a number of interesting challenges and opportunities. How did the different conditions prevailing in different parts of the landscape influence the recorded productivity of the land? And could the information on productivity recorded for government-owned land be generalised for the wider landscape? How enduring was the influence of these factors on the favourability of land for agriculture? Or in other words, could an improved understanding of the variability in land quality in the early modern landscape shed light on and pose useful questions for the study of earlier landscapes?

The work presented here attempts to provide the basis to address these questions by developing a GIS-based model that aims at understanding to what extent (if any) topographic and cultural factors could have influenced the agricultural quality recorded in the cabreo, so allowing the historic information to be generalised for the entire landscape. The process required to achieve this also entailed some innovative applications of GIS and statistical methods to accommodate historical spatially-referenced data, which are also interesting from a methodological point of view. As a matter of fact, to the best of the authors’ knowledge, the present work is the first attempt at using cabreo data for the purpose of model building in a GIS environment.

## The study area

**Fig 1:**
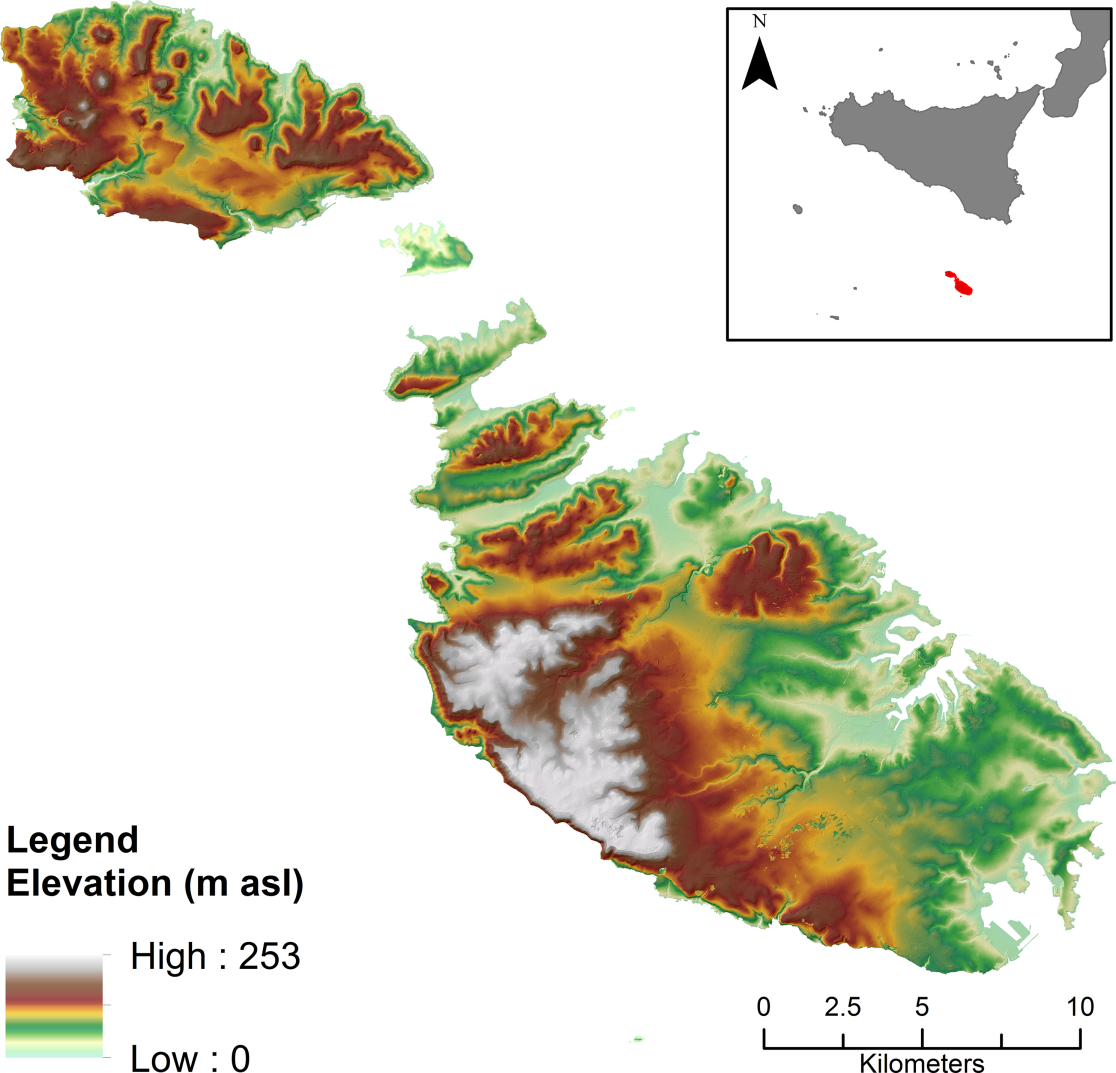
Maltese archipelago. Digital Terrain Model of the Maltese Islands, with inset showing its position relative to Sicily and southern Italy. Image created by the authors in ESRI’s ArcGIS 10.1; no copyrighted material was used.

The Maltese archipelago is located in the central Mediterranean, about 90 km south of Sicily (Fig 1).

The combined surface area of the archipelago is a mere 316 square km, largely made up by the two principal islands of Malta and Gozo. The present study is focussed on Malta, the largest island in the group. In spite of the small size of the island, the landscape is highly varied and fragmented. The northwest of Malta is characterised by a series of parallel ridges, which are separated by sheltered valleys that have been prime agricultural land at least since the early modern period. The west of the island is characterised by windswept uplands, while the centre and southeast are made up of gently rolling hills that are rather more favourable for agriculture. Several of the factors that may influence land quality, discussed in detail below, are evidently enduring features of the landscape, which may have also influenced land use in much earlier periods.

## Materials: Cabreo data and GIS

**Fig 2:**
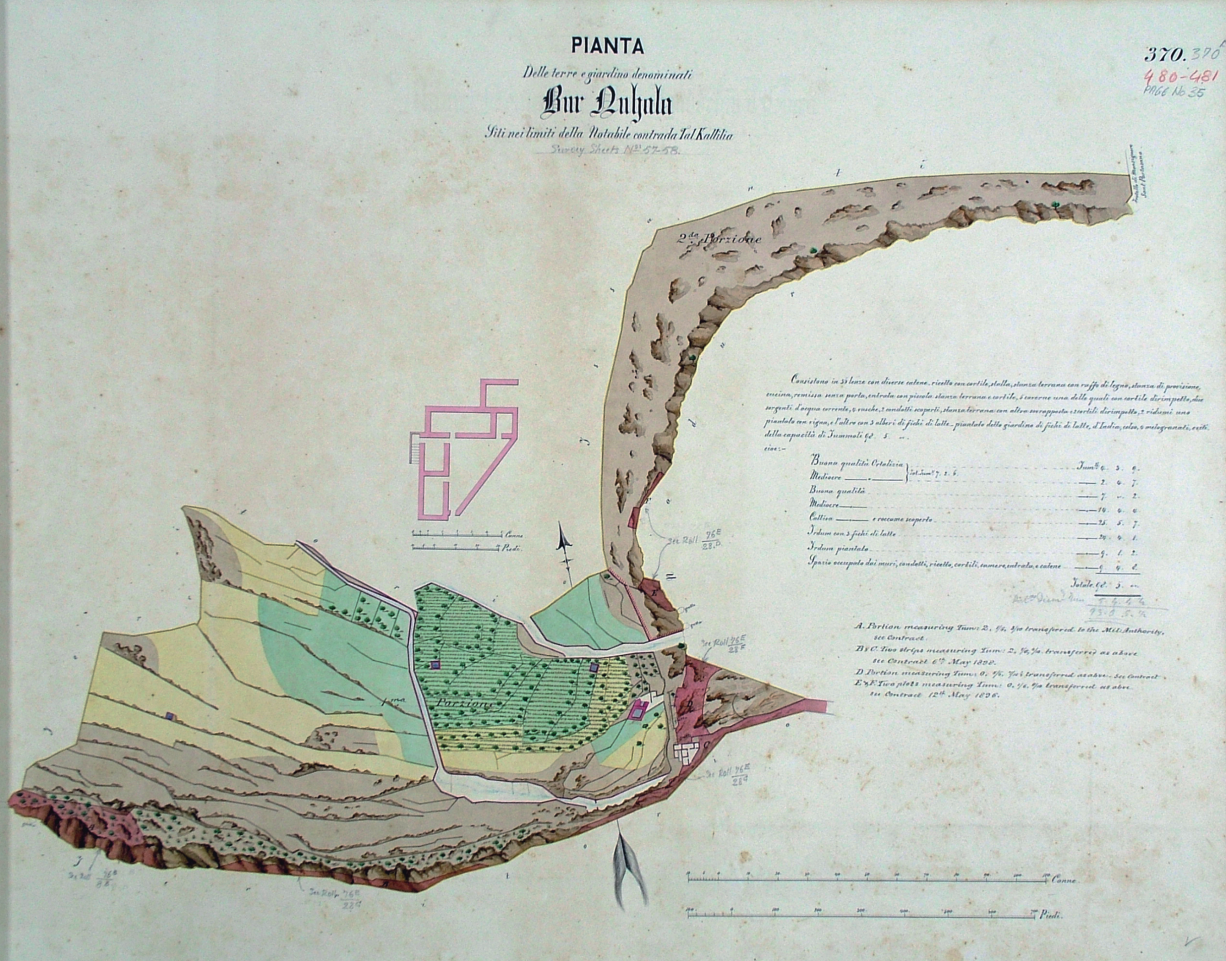
Cabreo map. Example of a 1800s cadastral map (courtesy of the National Archives of Malta) showing the extent and features of a state-owned parcel. Colours indicate different agricultural qualities. A hand-written legend is at the right-hand side of the map and provides a description of features such as water reservoirs, crop types, and presence of farmhouses and animals. See also Fig 3. Reprinted under a CC BY license, with permission from Dr Charles Farrugia (CEO and National Archivist, National Archives of Malta).

Cabreo maps [6,7] were made available in digital format (.*tiff* files) through the courtesy of the National Archives of Malta. The material dates to the mid-1800s and documents state-owned properties. The parcels of land are different in size and were consistently shaded in different colours (Fig 2).

After careful examination, it has been ascertained that colours correspond to different classes of agricultural productivity quality, as devised by the *agrimensori* (land surveyors) who were in charge of the creation of the cadastral maps [8]. Land productivity was either assessed on the basis of written records provided by the landowners or estimated by the land surveyors themselves. Through a quite complex calculation of the ratio between the amount of crops sown and that eventually harvested [9], the land surveyors were able to classify the productivity by means of a 5-tiered scheme, which was employed in the production of the cadastral maps. Green was consistently used for parcels whose agricultural yield was described as good, while dark brown featured parcels with an exposed rocky bank and labelled as bad. The full qualitative scale used in the cabreo ranges as follows, in descending order: *buona* (good), *mediocre* (mediocre), *cattiva* (bad), *inferiore* (lower), *infima* (lowest). Besides the key to the colour classification, an accompanying hand-written caption at the margin of each map recorded information about the type of crops grown, the presence of farmhouses and stables, the number and types of water facilities, and the presence of animals.

**Fig 3:**
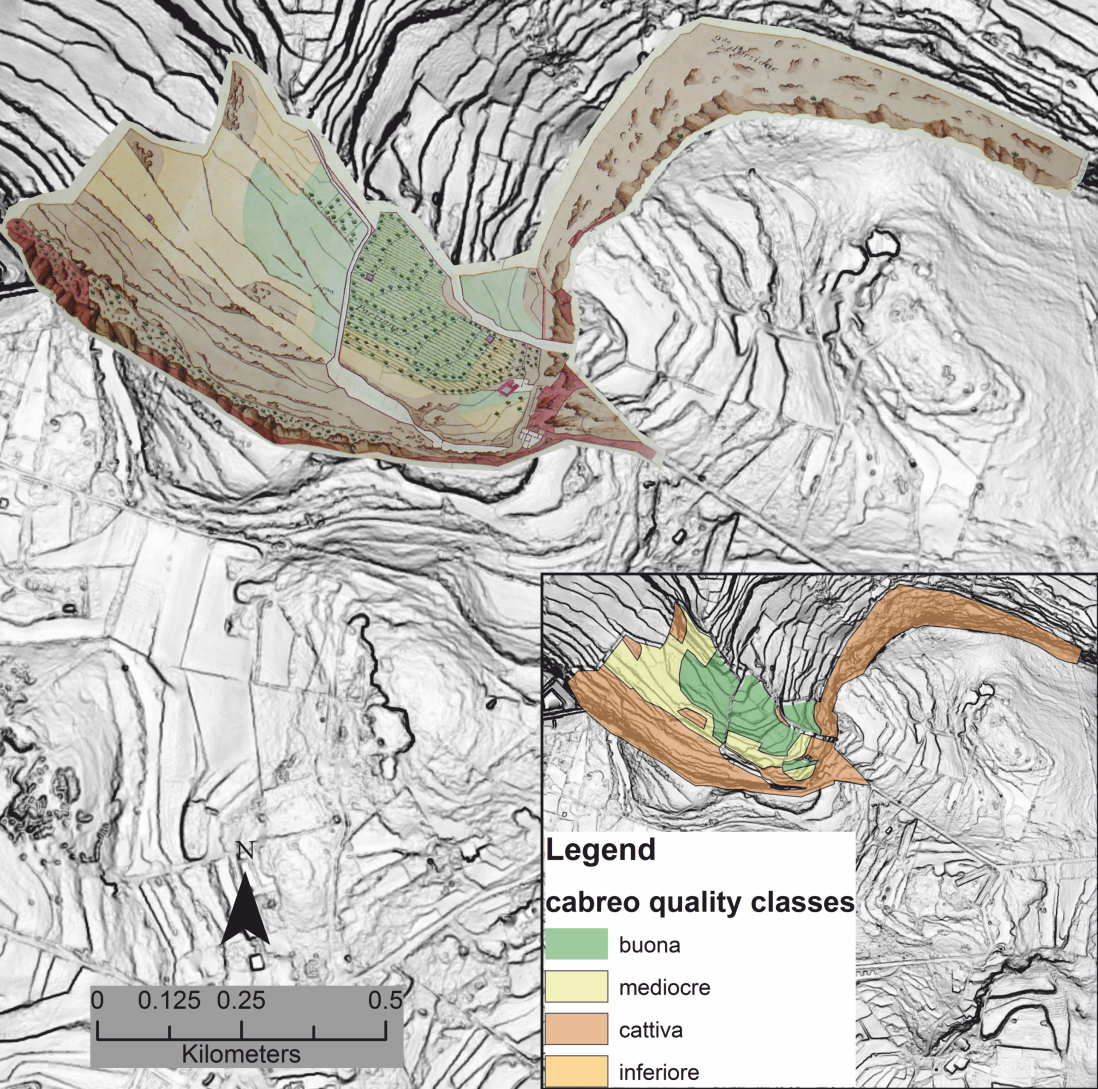
Cabreo maps georeferencing and digitization. Example of cadastral map georeferencing and digitization in GIS. The digitized version of the map is in the inset. Polygons represent sectors recorded as having different agricultural qualities. Quality ranges from *buona* (good, in Italian) to *inferiore* (inferior, in Italian). Image created by the authors in ESRI’s ArcGIS 10.1; no copyrighted material was used (see also Fig 2).

As a preliminary step, the dataset has been scrutinized in order to have a general understanding of the quality of the documentation itself. The whole dataset comprised 550 parcels, including maps of (*a*) extremely small tenements, (*b*) tenements that no longer exist due to modern urbanization, and (*c*) isolated buildings or farmhouses. Unfortunately, some of the images (*d*) showed colours that varied from those in the colour code noted above for land productivity quality. After excluding the maps falling in the mentioned four mentioned categories (*a-d*), the total sample of maps remaining was 250. Due to time constrains, and since maps were to be georeferenced and digitized, it was decided to draw a more manageable sub-sample in which maps from each of the three principal geographical regions of Malta could have the same probability of being chosen (namely, the low-lying hills and plains of south-east to central Malta, the parallel ridges and valleys in the northwest of the island, and the uplands in the western region). Random sampling was performed, stratified across the three regions, and 20 random maps for each macro-area were thus obtained. The fraction of the sub-sample relative to the parent dataset was arbitrarily set at 25%. The total area covered by the cabreo sub-sample is 6.70 sq km, corresponding to 2.72% of the area of the island of Malta, the largest island in the Maltese archipelago (246 sq km). The percentage rises up to 3.56% if the extent of areas urbanized today (58 sq km) is subtracted from the total are of the study region. The maps have been given spatial references (using ESRI’s ArcGIS 10.1) against georeferenced 1940s survey sheets used as base map (scale: 6 inches to 1 mile), and have been digitized by using polygons (Fig 3).

**Fig 4:**
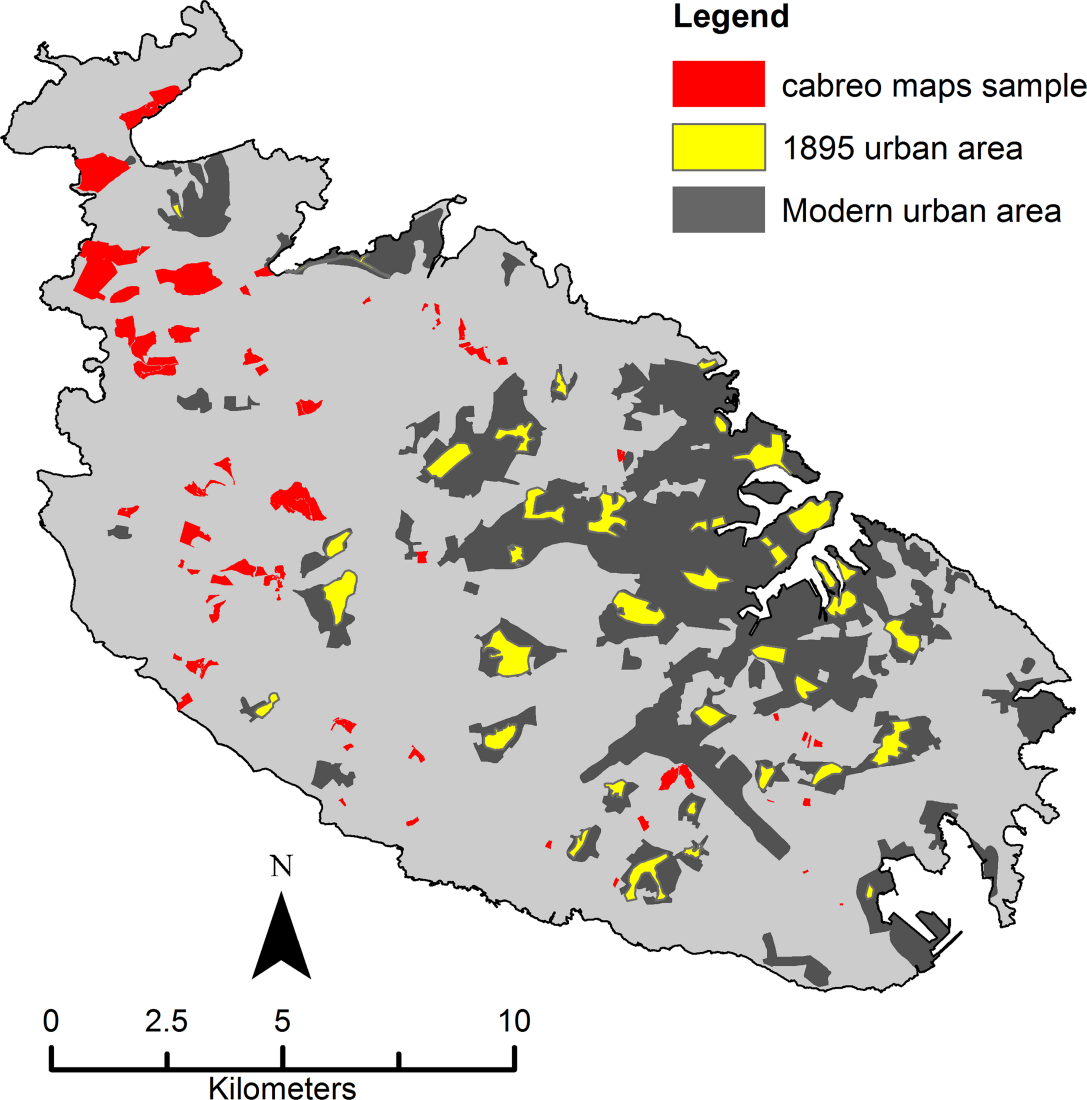
Cabreo maps sample. Sample of cabreo maps used for model building. Urban areas, in their 1895 and modern extent, are also shown. Image created by the authors in ESRI’s ArcGIS 10.1; no copyrighted material was used.

Different information has been stored in the attribute table of the polygon layer, the most significant for the purposes of the present study being the agricultural quality class registered for each parcel or part thereof. A total of 318 polygons were used (Fig 4).

## Method

### Logistic regression

Logistic regression (hereafter LR) is widely used in different research fields, spanning from social to hard sciences [10–16]. It finds extensive use in GIS-based studies [10–11,17–19] since it allows modelling of the relation between a nominal dependent variable and independent variables (i.e., predictors) of different types (nominal and/or continuous). The reader is referred to the existing literature for an in-depth treatment of the topic [20–22]. LR makes it possible to estimate the probability that a particular outcome of a dependent nominal variable (*y*) will occur based on information from one or more predictors (*x*_*m*_). The technique ultimately finds the equation that best predicts the probability *p* of getting a particular value of *y*, with *p* taking values from 0.0 to 1.0. If *m* is the number of predictors, the general form of the logistic regression model is:

$$p=\frac{e^{\beta_{0}+\beta_{1}x_{1}+\beta_{2}x_{2}+\ldots +\beta_{m}x_{m}}}{1+e^{\beta_{0}+\beta_{1}x_{1}+\beta_{2}x_{2}+\ldots +\beta_{m}x_{m}}}$$

Unlike the least-squares method used in linear regression, logistic regression finds the intercept (*β*_0_) and slopes (also termed logistic regression’s coefficients; *β*_1_, *β*_2_ … *β*_m_) of the best-fitting equation by means of the maximum-likelihood method, which is a computer-intensive technique that finds *the values of the parameters under which you would be most likely to get the observed results* [23]. The LR equation consists of values of the predictors plus weights estimated by the model to predict the outcome of the dependent variable [24]. Once logistic regression has been run, and the intercept and coefficients have been found, it is possible to calculate the probability of the outcome of *y* by plugging those parameters and any known value of the predictors into the logistic regression equation.

The model’s coefficients can be meaningful interpreted once they are exponentiated and thus expressed in terms of odds ratio [22]. An exponentiated coefficient of 1 leaves the odds for the positive outcome of the dependent variable unchanged, while a coefficient greater or smaller than 1 increases or decreases the odds respectively. For instance, if the presence of, say, a landslide is modelled as dependent on the terrain slope, and assuming that the latter has an estimated coefficient of 0.1871, its odds ratio is 1.206 (i.e., *e*^0.1871^). This indicates that a 1-unit increase in slope increases the odds of landslide by a factor of 1.206. In case of categorical predictors, the following interpretation holds. Let’s assume that the presence of landslides is modelled as dependent also on the type of soil, and the latter categorical predictor has three levels, soil A, B, and C. Usually, one of the levels is used as reference category and used as baseline for comparison. In other words, assuming that the reference level is soil A, an exponentiated coefficient of, say, 1.5 for soil B and of 0.50 for soil C indicate that B increases the odds for landslide by 1.5 relative to soil A, while C decreases the odds by 0.50 relative to the same reference level.

In this study, the analysis of the area under the ROC curve is used to assess the discriminatory power of the model [20,25–27]. It plots the proportion of cases correctly classified as a positive outcome of the dependent variable (*sensitivity*) versus the proportion of cases incorrectly classified as a negative outcome (*1 minus specificity*) for the entire range of possible cut-off points on the model’s probability. The area under the ROC curve (AUC) provides an overall measure of the model’s ability to discriminate between the two outcomes of the dependent variable [20] in the dataset on which the model has been trained: the more the curve deviates from 45°, the higher is the model’s power. As a rule of thumb, the AUC value can be classified as follows [20]: discriminatory ability *no better than chance* (0.5), *poor* (0.5–0.7), *acceptable* (0.7–0.8), *excellent* (0.8–0.9), *outstanding* (0.9–1.0).

### Rationale behind the choice of the model’s variables

A choice has been made as to what variables could be meaningfully used in this study. While, as touched upon earlier, the dependent variable is the quality of the land productivity as classified in the cabreo data, a critical choice needed to be made regarding the use of the whole cabreo qualitative scale. It was decided to collapse the cabreo classes into two broad ones, i.e. optimal (corresponding to the *good* class) vs. non-optimal quality (comprising the *mediocre*, *bad*, *low*, and *lowest* classes). This dichotomization was considered appropriate to this study’s goal of understanding which predictors are likely to have contributed to optimum agricultural yield. Of course, the model could be further refined in future stages of this research, making use of the whole cabreo classification, or collapsing categories in a different fashion.

**Fig 5:**
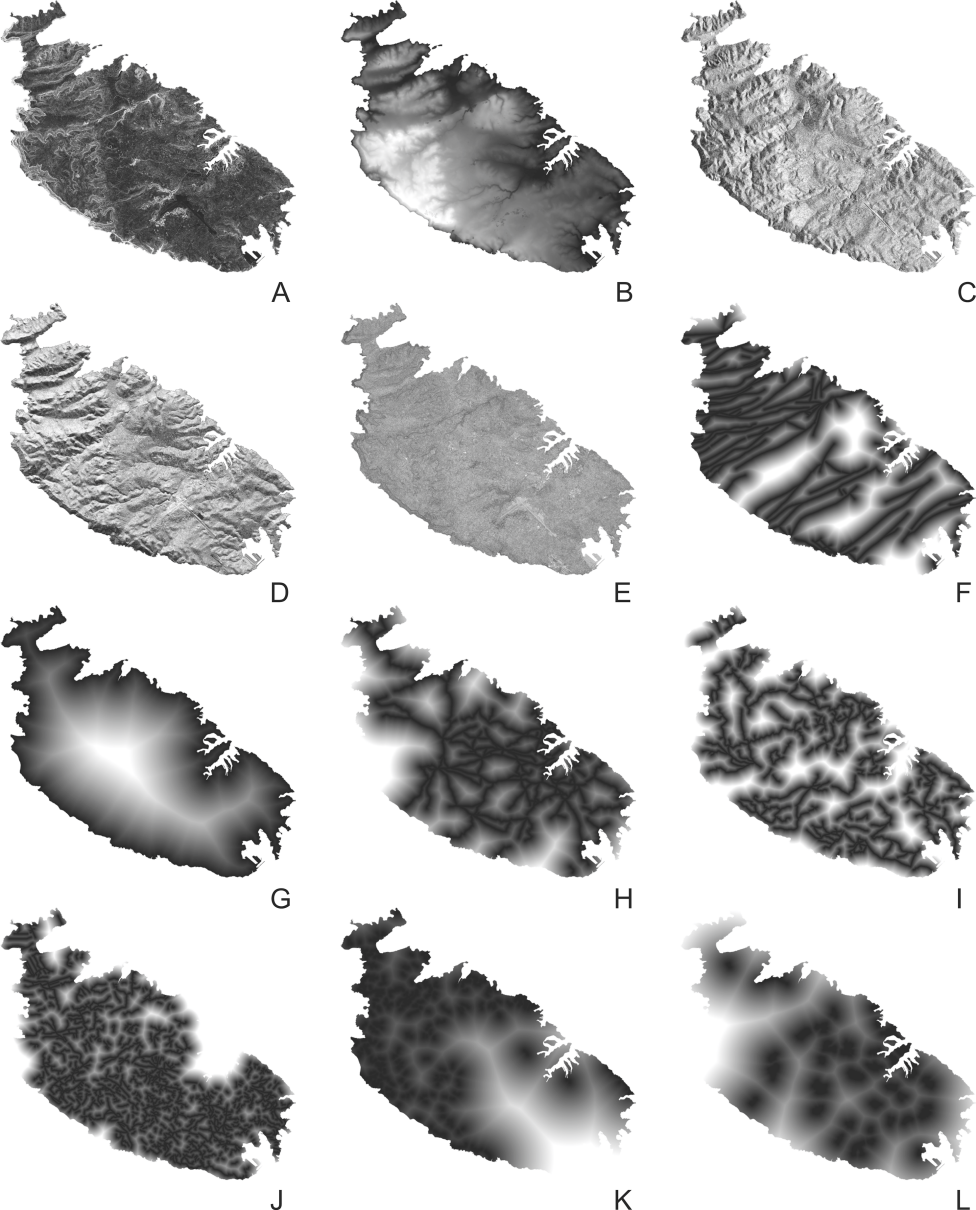
Logistic regression model: Predictors. Selection of predictors used in the cabreo model: (A) slope, (B) elevation, (C) sine of the aspect, (D) cosine of the aspect, (E) Topographic Wetness Index, (F) distance to the nearest geological fault-line, (G) distance to the coast-line, (H) distance to the nearest main, (I) secondary, (J) minor 1895 road, and (K) to the nearest footpath, (L) distance to the nearest 1895 urban area. See also Table 1. Image created by the authors in ESRI’s ArcGIS 10.1; no copyrighted material was used.

A total of 17 predictors have been used in the model. We must acknowledge that modelling agricultural suitability is not a simple task and there is no unique set of criteria to be taken into consideration when studying agricultural potential [28]. A complex interplay between at least socio-cultural, economic, climatic, environmental, and topographic factors may indeed affect the inherent properties of the land in the context of its use as well as of its abandonment [28,29]. While they cannot be considered the only determinants of agricultural suitability, the predictors used in this study were deemed useful on the basis of the literature review and data availability (Table 1 and Fig 5).

**Table 1:** Dependent and independent variables used for the logistic regression model.

Variables	Type	Levels	Importance
*Dependent variable*			
Cabreo agricultural quality	categorical (binary)	non optimal	
		optimal	
*Independent variables*			
Slope (degrees)	continuous		water flow velocity, moisture, soil depth
Elevation (m)	continuous		climate, water drainage, moisture
Aspect (sin)	continuous		insolation, evapotranspiration, moisture
Aspect (cos)	continuous		insolation, evapotranspiration, moisture
Curvature-planform	continuous		water convergence
Curvature-profile	continuous		water flow acceleration/deceleration, erosion
Topographic Wetness Index	continuous		soil water content
Distance to fault lines (km)	continuous		fresh water availability
Distance to coastline (km)	continuous		sea-spray, salt-laden air
Soil	categorical	brown rendzinas	hydraulic conductivity, nutrients management
Soil	categorical	xerorendzinas	hydraulic conductivity, nutrients management
Soil	categorical	carbonate raw	hydraulic conductivity, nutrients management
Soil	categorical	terra rossa	hydraulic conductivity, nutrients management
Soil	categorical	Iklin-Tad Dawl*	hydraulic conductivity, nutrients management
Distance to main roads (km)	continuous		land accessibility
Distance to second. roads (km)	continuous		land accessibility
Distance to minor roads (km)	continuous		land accessibility
Distance to footpaths (km)	continuous		land accessibility
Distance to urban areas (km)	continuous		land accessibility
X coordinate (m)	continuous		accounting for spatial auto-correlation
Y coordinate (m)	continuous		accounting for spatial auto-correlation

*Reference category

While factors such as salinity and sodicity, soil texture, soil depth, winds, precipitation, and climate variability are likely to influence land quality in terms of suitability for agriculture [28,30–33], the first eight variables listed in Table 1 have been considered potentially important for modelling agricultural suitability for their connection with soil moisture and water availability. Indeed they are widely used in the available literature [28,34–36]. Moisture and availability of water are key factors for the understanding of the distribution patterns of vegetation and human activities in the Maltese archipelago [37,38]. As the table summarizes, some of the predictors affect the water flow, which influences the processes of soil erosion and deposition, which in turn affect soil depth and fertility. Terrain attributes can contribute to predict a significant portion of the spatial organization of sediments and microclimate [39], soil moisture [40], and soil properties [41]. As different authors point out [26,31–33,42–48], variation in slope, aspect, relative elevation, and curvature, affect the distribution of moisture near the land surface, with slope influencing infiltration, drainage, and runoff. Steeper slopes are likely to be drier than flat areas due to lower infiltration rates and higher surface runoff, and are also likely to have shallower soils. The amount of solar energy is also higher on steep slopes, since the quantity of solar radiation per unit area of the land surface decreases as the slope decreases. Steeper slopes are obviously more difficult to cultivate relative to more gentle sloping terrains [28,49]. Elevation is associated with lower temperatures and lower moisture content [28,42,44]. In fact, elevation can affect water retention since terrains at greater elevation may have more soil water draining down and are subject to receive less water from upslope [44,46]. Aspect (i.e., slope orientation) influences solar radiation, and hence evapotranspiration, soil moisture, and soil nutrients [41,42,46,50]. Slopes with different orientation are differentially subject to sunlight and prevailing winds. For these reasons, aspect is taken into consideration as an assessment criterion for the selection of the land to be used for agriculture [28,49]. Curvature, which can be further characterized as planform (i.e., perpendicular to the slope direction) and profile (i.e., in the direction of the slope) [51], influences convergence/divergence and acceleration/deceleration of rainwater flow since the latter is related to the convexity or concavity of the terrain. Where acceleration of flow occurs (convex profile curvature) erosion will be higher and soil water content lower, whereas in areas with concave profile curvature erosion is lower [52] and deceleration of flow allows water to accumulate [43]. A Topographic Wetness Index (TWI) has also been taken into account. A combination of parameters such as flow-accumulation and slope of a given cell was used to give an indication of the tendency of water to accumulate at any point of the area under study [43,51]. High values correspond to converging flat terrains, while low values are typical of steep and diverging areas [53–55].

The Euclidean distance from the main geological faults has been used as a proxy for fresh water availability. Fault lines may facilitate infiltration of rainwater into the ground [56], also providing fissures and micro-fractures along which the water retained by geological clayish layers may escape [57]. The relation between springs and fault lines has been tested in a preliminary step of the present study. The distance to the nearest fault line of 49 *Ghajn* toponyms (place-names indicating the actual presence of a spring; located on the basis of an earlier work [58]) has been compared to the distance of 49 random points. The toponyms show a tendency to be closer to the fault lines (mean distance: 251 m) compared to random points (mean distance: 423 m). The mean difference in distance (172 m) is statistically significant (permuted *p* value based on 999 permutations: 0.011). As for the distance from the coastline, it has been considered as a predictor since lands close to the coast could be more subject to the negative effects of sea-spray and salt-laden air [37].

**Fig 6:**
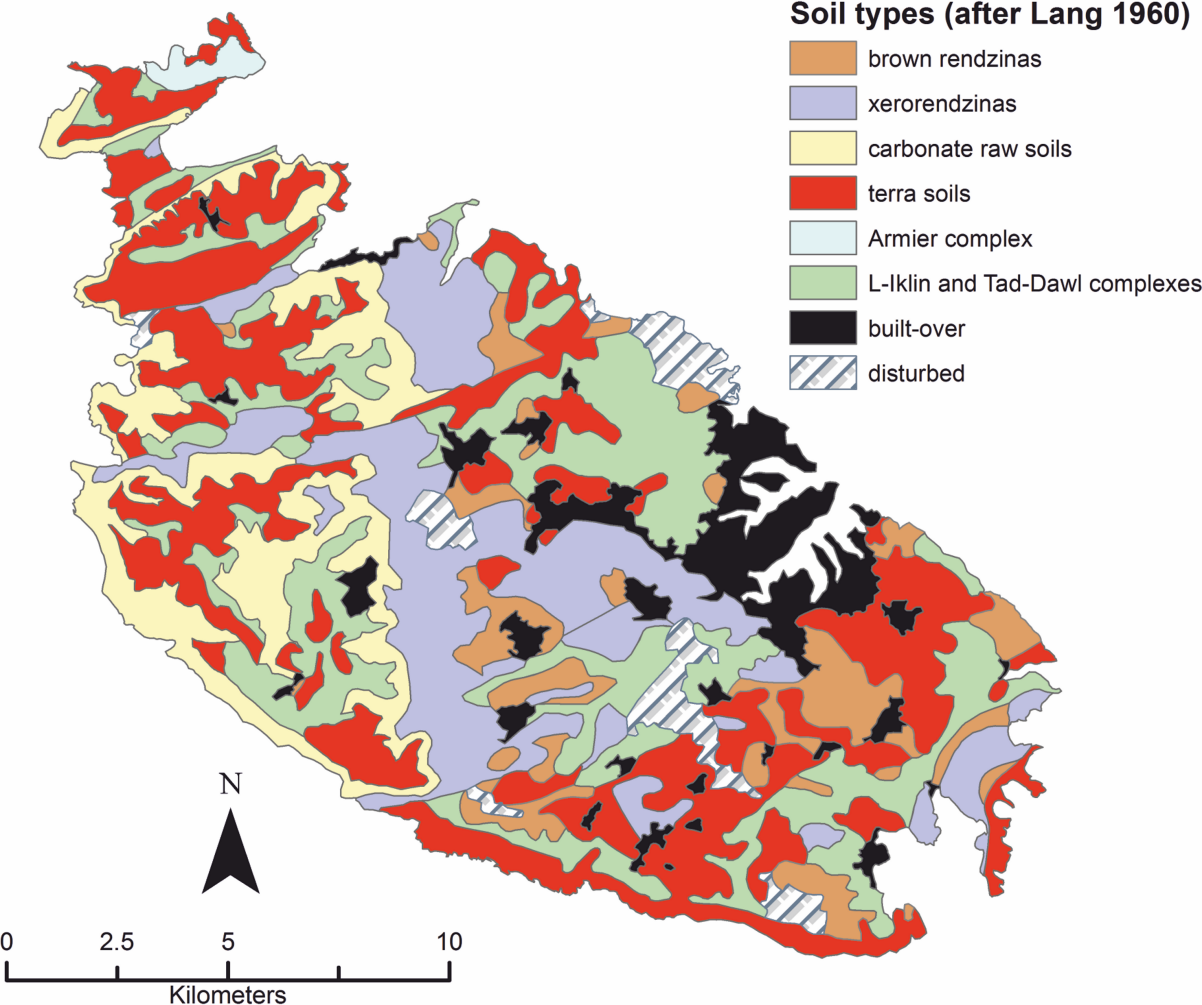
Soil types. Extent and distribution of soil types in Malta. Produced by georeferencing and digitizing the map published in [60]. Image created by the authors in ESRI’s ArcGIS 10.1; no copyrighted material was used.

Soil type (Fig 6) has been chosen as a predictor (comprising 5 levels) since soils provide essential nutrients to plants and their different physical characteristics allow water and air to infiltrate, roots to explore, and biota to thrive [59].

Soils vary in their ability to drain and retain water and nutrients at a different pace, and are therefore differentially suitable for agriculture. The soil classification used for this study is that devised by Lang [60,61] in his detailed analysis of the soils of the Maltese Islands. He provided descriptions of the soils and of their distribution, mapping differences in chemistry, physics, and biology. His work was intended as an aid to agricultural planning and development in the study area. Interestingly, he also provided some remarks on the agricultural suitability of the different soil types, basing his observations on his first-hand knowledge of the Maltese man-made landscape [60,62].

**Fig 7:**
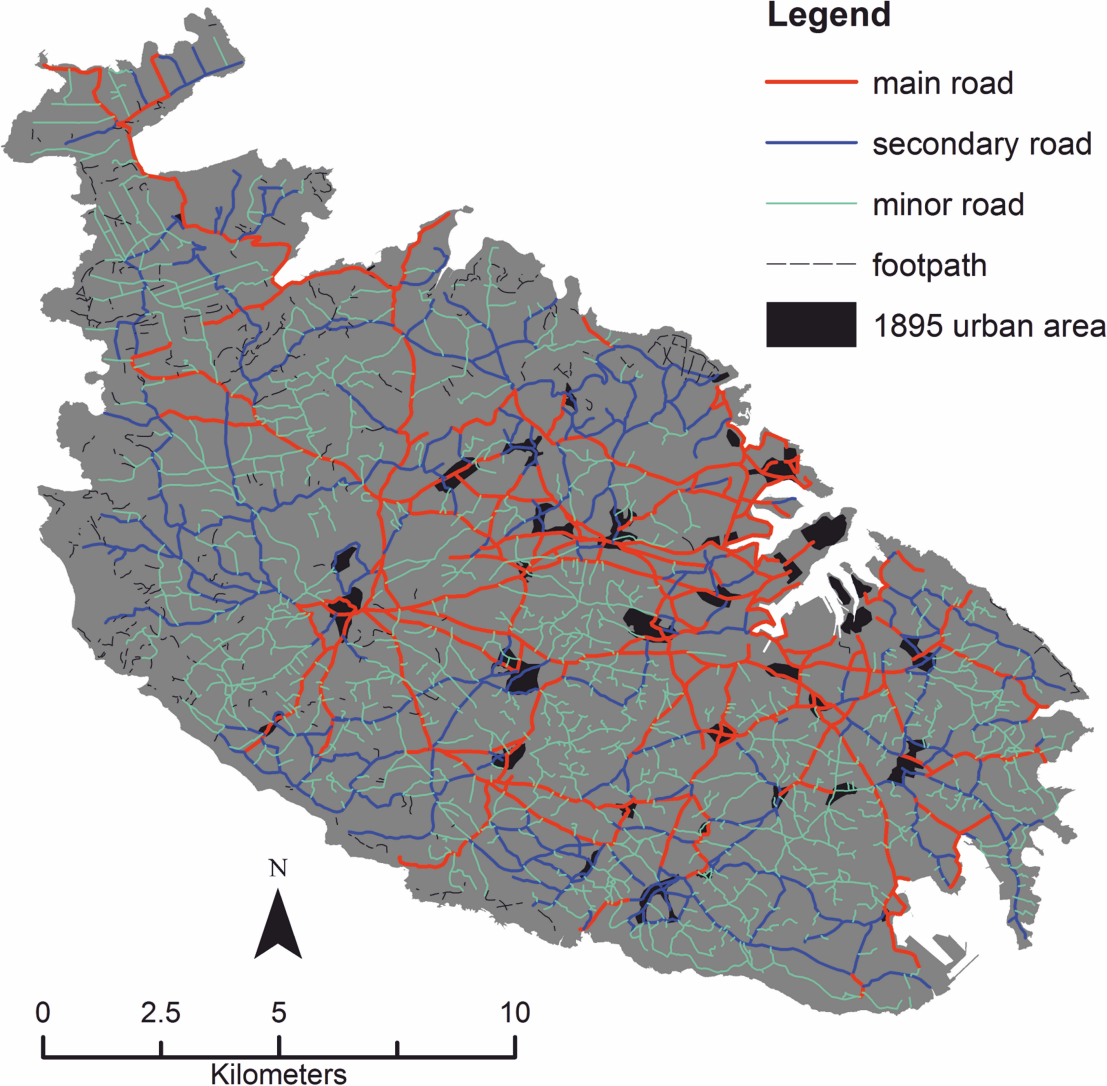
1895 road network in Malta. Road network in Malta in 1895, broken down by road type. Footpaths and the 1895 urban area are also shown. Image created by the authors in ESRI’s ArcGIS 10.1; no copyrighted material was used.

In order to assess the possible influence of land accessibility on the agricultural quality, the distances to the nearest road (classified as main, secondary, or minor) and to the nearest footpath have also been taken into account as predictors (Figs 5 and 7).

Road networks may provide an important driving force in shaping the relation between human activity and spatial phenomena, such as urban growth [63], forest clearance and agricultural abandonment [12,64,65], land use changes and vegetation dynamics [66,67], and the spatial organization of land use [68]. The use of these predictors allows the importance of this cultural factor to be compared to that of environmental and topographic ones [64]. For the same reason [68], the distance to the nearest urban area (as it stood in 1895) has also been included as a predictor. Finally, the geographic coordinates have been entered as predictors (see later on).

### Modelling strategy and sample size

Each independent variable was entered into ArcGIS (10.1) as a raster layer. The elevation layer is a Digital Terrain Model (1 m cell size) derived from LiDAR [69] data made available through an agreement signed between the University of Malta and the former Malta Environment and Planning Authority in 2013. Slope, aspect, and curvature layers were obtained from the DTM by using the appropriate tools in ArcGIS *Spatial Analyst*. Aspect was split into two components, aspect-cosine and aspect-sine [46,51,70], producing values from 1 to -1. North-facing slopes have aspect-cosine tending to 1, whereas south-facing ones tend to -1. East-facing slopes have aspect-sine tending to 1, whereas west-facing slopes will tend to -1. The TWI raster was produced in ArcGIS using the *Geomorphometry and Gradient Metrics* toolbox created by Jeffrey Evans and colleagues [71]. The raster layers expressing the distance from the coastline, geological faults, road types, and 1800s urban areas, were generated on the basis of vector data created by digitazing those features against different survey sheets, which have been preliminarily georeferenced. The 1993 geological map of the Maltese Islands (scale 1:25,000) [57] has been used for digitizing the fault lines, the geological formations (which have not been used as predictors, for the reasons explained later on in this paragraph), and the coast-line. For the road network and urban areas, the map of the Island of Malta (sketched and compiled by captain E. M. Woodward, Leicestershire Regiment D.A.A.G.) has been used (scale 1:21,120), dating to 1895. Finally, Lang’s map (scale 1:31,680) charting the distribution and extent of soil types in Malta has been used to feed soil information into GIS.

A set of random points (n = 16,643) with a minimum spacing of 20m was generated within the polygon layer representing the cabreo maps. Each point has been used as a sampling location [11] and has been given values of both the dependent variable and the predictors. The spacing of the points was chosen to alleviate spatial autocorrelation [72], which poses issues to traditional statistical methods in terms of inference, coefficients estimation, and assessment of the relative importance of predictors [72–76]. Two additional steps were taken beside the 20m points spacing. First, a 25% random sub-sample of points (n = 3,897) was drawn from the larger universe of sampling points to further increase the between-points distance relative to the full dataset [63]. The subjective choice of the 25% fraction was made in an attempt to find a balance between: a) dealing with a more manageable sample, which could also be less prone to assure statistical significance to otherwise small and unimportant effects, just as a result of the increase in power due to the very large sample size [20,22]; b) assuring an adequate sample size to properly perform LR (as discussed later on). Secondly, the geographic coordinates of each sampling point have been entered in the model as predictors. Besides using an auto-covariate term as predictor [72–74,76,77], adding geographic coordinates as extra predictors is well documented in the literature as a method to alleviate spatial autocorrelation [63,78–83]. The presence of spatial autocorrelation in the model’s residuals has been nonetheless tested [84–88] to formally check if the fitted model and the estimated coefficients can be considered reliable.

Predictors have been preliminarily checked for the presence of a strong correlation (i.e., collinearity) among them [89]. Pearson’s *r* has been calculated between pairs of predictors, and 0.70 has been considered as a critical threshold [89–91]. Table 2 shows that there is no critical collinearity among predictors.

**Table 2:** Pearson correlation among model’s predictors.

Predictors	1	2	3	4	5	6	7	8	9	10	11	12	13	14	15	16
(1) elevation	**-**															
(2) slope	0.056	**-**														
(3) aspect (cos)	-0.072	0.047	**-**													
(4) aspect (sin)	0.073	-0.045	-0.072	**-**												
(5) curvature-planform	0.020	0.058	0.025	0.008	**-**											
(6) curvature-profile	-0.005	-0.043	0.007	0.002	-0.629	**-**										
(7) TWI	0.000	-0.473	-0.020	0.007	-0.295	0.192	**-**									
(8) coast distance	0.534	-0.029	-0.105	0.129	-0.009	-0.001	0.058	**-**								
(9) fault line distance	-0.019	-0.043	0.138	-0.018	0.000	-0.007	0.006	0.122	**-**							
(10) urban areas distance	0.114	0.053	0.036	-0.076	0.037	0.004	-0.050	-0.337	-0.375	**-**						
(11) main roads distance	0.462	-0.003	-0.051	0.015	-0.005	0.008	0.034	0.056	-0.093	0.148	**-**					
(12) minor roads distance	-0.162	0.056	-0.083	0.089	-0.024	-0.002	-0.031	-0.184	-0.109	0.141	-0.131	**-**				
(13) secondary roads distance	-0.297	0.031	0.083	-0.035	0.011	0.000	-0.012	-0.305	-0.013	-0.002	0.100	-0.114	**-**			
(14) footpaths distance	0.067	-0.010	-0.002	0.051	-0.010	-0.008	0.032	0.448	0.535	-0.471	-0.245	-0.068	-0.183	**-**		
(15) X	0.126	-0.063	-0.022	0.031	-0.006	-0.011	0.063	0.531	0.360	-0.529	-0.304	-0.094	-0.286	0.684	**-**	
(16) Y	-0.658	0.012	-0.030	-0.017	-0.004	0.007	-0.061	-0.596	-0.281	0.256	-0.063	0.260	0.301	-0.636	-0.712	**-**

Variance Inflation Factor was also considered since no pair of predictors is critically correlated but there are several variables tied by interdependencies [90]. VIF shows *how much the variance of the coefficient estimate is being inflated by multicollinearity* [90]. In our case (Table 3), no predictor has a VIF larger than 10, which is the critical value suggested in the literature [89,90].

**Table 3:** Predictors’ variance inflation factor.

Predictors	VIF
elevation	5.816
slope	1.343
aspect (cos)	1.095
aspect (sin)	1.056
curvature-planform	1.769
curvature-profile	1.661
TWI	1.435
coast distance	2.377
fault line distance	1.644
urban areas distance	1.766
main roads distance	1.669
minor roads distance	1.239
secondary roads distance	1.311
footpaths distance	4.897
X	6.753
Y	8.003

Geology has not been used as predictor since, in a preliminary step of the present study, it proved (as expected) strongly correlated to soil types (Table 4).

**Table 4:** Cross-tabulation of Maltese soil types (after [60]) (rows) against geological formation (columns); the association is as expected strong and significant (see text).

	Blue Clay	Globigerina	Lower Coralline	Upper Coralline	
**Brown Rendzinas**	1	131	0	10	*142*
**Carbonate Raw Soil**	526	141	3	308	*978*
**L-Iklin-Tad-Dawl**	8	26	4	293	*331*
**Terra Rossa**	39	184	0	1912	*2135*
**Xerorendzinas**	24	217	3	67	*311*
	*598*	*699*	*10*	*2590*	**3897**

This was assessed using chi-square test (chi-square: 2864.67, df: 12, p: <0.0001) and Pearson’s *phi* coefficient (0.86) [92].

As for sample size for LR [20], we followed Peduzzi et al. [93] who provide a guideline for calculating the minimum sample size: considering at least 10 observations per predictor, divided by the proportion of negative (‘non-optimal’ quality; n = 2,965) or positive cases (‘optimal’ quality; n = 932), whichever is smaller. In our case, the minimum size is 708 observations (i.e., 10x17:0.24). The sample used in this study is more than five times larger than the minimum necessary for a model with 17 predictors. The dataset on which the model has been built is available in a tab-delimited.txt file (which can be easily imported into any statistical program) provided as Supporting Information (S1 Text. Dataset).

### Predictors selection and model validation

The “best” model was selected via the backward stepwise procedure implemented by D. Rizopoulos’s *boot*.*StepAIC* R package [94]. While mixed opinions exist about stepwise procedures [20,95–97], the aim of such an approach is to isolate a parsimonious model. The principle of parsimony suggests avoiding models with unnecessary complexities, and to give preference to simpler models (in comparative terms) that explain the greatest amount of data variability with the lowest level of complexity [98,99]. The package implements the model selection devised by Austin and Tu [100], widely used in literature [101–104]. The assessment of how much the selected model is able to generalize outside the training data (i.e., model validation [20,27,105,106]) has been performed by means of internal validation, following the method described by Arboretti Giancristofaro-Salmaso [27], which has been implemented in *R* [107,108]. Further details about both procedures are provided as Supporting Information (S2 Text. Predictors selection and model validation).

## Results

Eleven predictors out of 17 candidates can be considered truly independent ones since they were selected in practically all the 1000 bootstrap resamples (Table 5).

**Table 5:** Percentage of times in which each candidate predictor was selected using the backward stepwise model selection in 1000 bootstrap samples, following the method devised by [100] and implemented in R by [94].

Predictors selected	%
coast distance	100
elevation	100
fault distance	100
footpaths distance	100
secondary roads distance	100
soils	100
X	100
Y	100
minor roads distance	99.9
slope	99.8
aspect (sin)	99.7
curvature-planform	48.2
TWI	35
curvature-profile	34.2
urban areas distance	21.6
aspect (cos)	18.1
main roads distance	18.1

The six excluded predictors (planform curvature, TWI, profile curvature, distance to the nearest urban area, cosine of the aspect, distance to the nearest main road) were selected only in tiny fractions of the samples, and their estimated coefficients were unstable, indicating that they do not contribute to the prediction of the outcome of the dependent variable (Table 6).

**Table 6:** Percentage of times in which each candidate predictor resulted having a positive or negative coefficient during the backward stepwise model selection in 1000 bootstrap samples.

Coefficients sign	+ (%)	- (%)
aspect (sin)	100	0
coast distance	100	0
minor roads dstance	100	0
soils = "Xerorendzinas"	100	0
soils = "Carbonate Raw"	99.5	0.5
TWI	98.57	1.43
curvature-planform	97.3	2.7
soils = "Brown Rendzinas"	97	3
curvature-profile	94.15	5.85
main roads distance	66.3	33.7
aspect (cos)	38.12	61.88
urban areas distance	34.26	65.74
soils = "Terra Rossa"	2.1	97.9
elevation	0	100
fault line distance	0	100
footpaths distance	0	100
secondary roads distance	0	100
slope	0	100
X	0	100
Y	0	100

**Fig 8:**
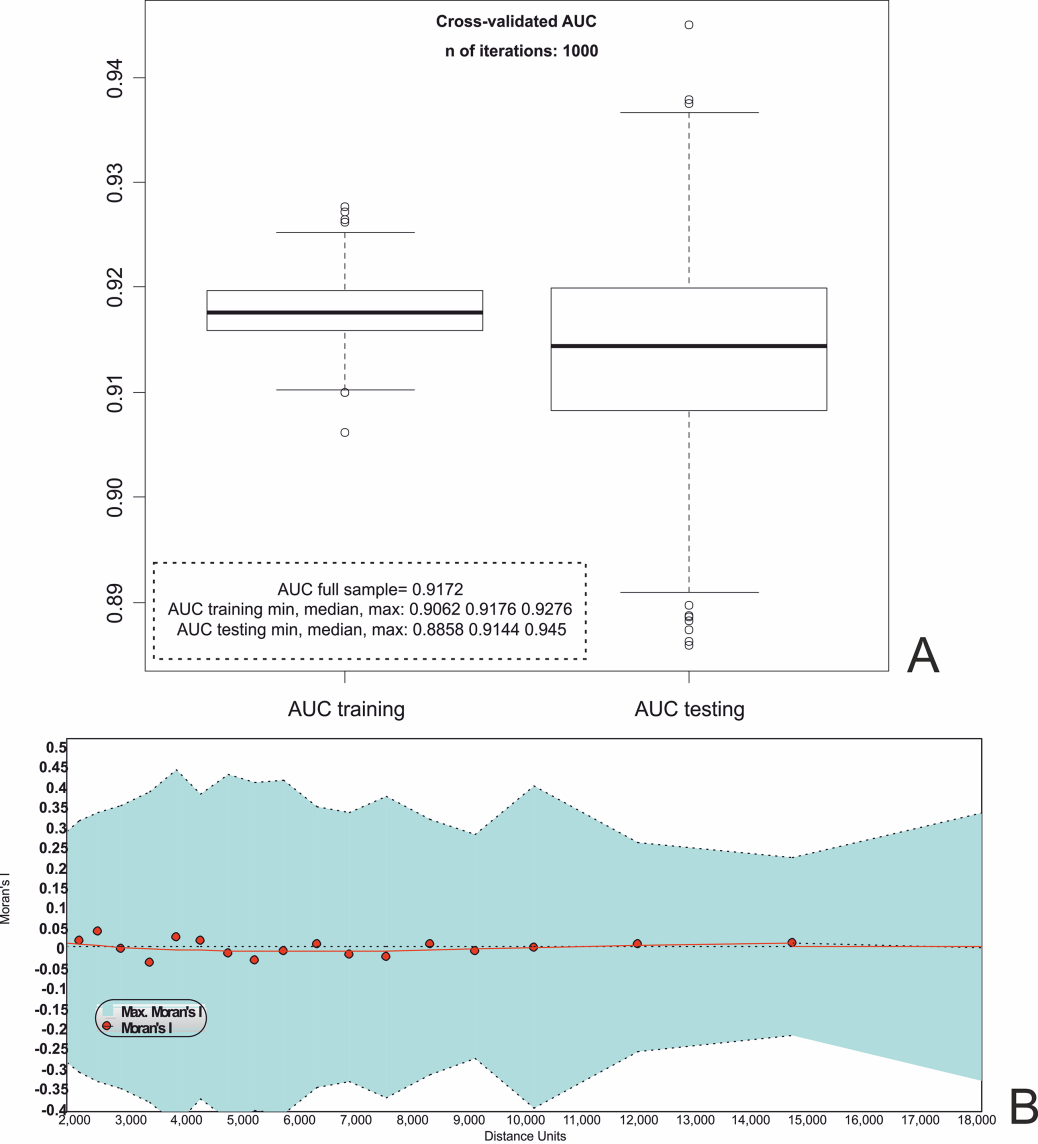
Logistic regression model diagnostic. (A) Internal validation following the procedure devised by [27] and implemented in R [108]; ‘AUC training’ provides an estimate of the performance of the model in the population of all the theoretical training samples; ‘AUC testing’ represents an estimate of the model’s performance on new and independent data. The latter indicates that the model excellently discriminates both on the original sample and outside it. (B) Spatial correlogram, returned by the SAM program (v. 4.0), showing a negligible spatial autocorrelation among the model’s residuals.

The model comprising the selected predictors is statistically significant: the *p* value of the difference between the *null* and the *full* model is well below 0.01, indicating that the predictors significantly affect the outcome of the dependent variable. The discriminatory power of the model can be considered excellent/outstanding, according to the 5-tiered scale described earlier: the AUC value is 0.92. As for model validation (Fig 8A), the fitting distribution of AUC is excellent, with a median value which is practically identical to the AUC for the original full dataset, and a minimum value of 0.91 which still points to an excellent/outstanding discriminatory power.

The validation distribution of AUC is excellent, with about 50% of the values falling between 0.91 and 0.92, and nearly 25% of them being larger than 0.89. This indicates that the model excellently discriminates both on the original sample and outside it. No spatial autocorrelation proves present in the model residuals. The spatial correlogram returned by the SAM 4.0 program [109] shows that the values of Moran’s I are extremely close to 0 along all the 22 distance classes (Fig 8B). Their statistical significance is just the result of the very large sample size.

The details of the fitted model are summarized in Table 7.

**Table 7:** Fitted logistic regression model results.

*Fitted LR model*						
*Sample size*	3897					
*Cases with Y = 0 (non-optimal)*	2965 (76.08%)					
*Cases with Y = 1 (optimal)*	932 (23.92%)					
*Overall Model Fit*						
*Null model -2 Log Likelihood*	4287,54					
*Full model -2 Log Likelihood*	2392,261					
*Chi-squared*	1895,28					
*DF*	14					
*Significance level*	P < 0.0001					
*Predictors Coefficients*						
* *	Coefficient	Std. Error	Wald	P	Odds Ratio	OR 95% CI
*elevation*	-0.041	0.002	316.848	<0.0001	0.960	0.956–0.965
*slope*	-0.032	0.006	28.514	<0.0001	0.969	0.957–0.980
*aspect (sin)*	0.352	0.083	17.815	<0.0001	1.422	1.207–1.674
*coast distance*	0.882	0.064	190.840	<0.0001	2.415	2.131–2.736
*fault line distance*	-2.391	0.334	51.342	<0.0001	0.092	0.048–0.176
*soils = "Brown Rendzinas"*	0.838	0.377	4.952	0.0261	2.313	1.111–4.882
*soils = "Carbonate Raw"*	0.964	0.309	9.702	0.0018	2.622	1.429–4.808
*soils = "Terra Rossa"*	-0.777	0.304	6.553	0.0105	0.460	0.254–0.8334
*soils = "Xerorendzinas"*	2.223	0.277	64.392	<0.0001	9.232	5.365–15.888
*secondary road distance*	-1.680	0.207	66.021	<0.0001	0.186	0.124–0.279
*minor road distance*	1.584	0.385	16.931	<0.0001	4.876	2.292–10.370
*footpath distance*	-0.897	0.153	34.534	<0.0001	0.408	0.302–0.550
*X*	0.000	0.000	35.852	<0.0001	1.000	0.999–0.999
*Y*	-0.001	0.000	300.619	<0.0001	0.999	0.999–0.999
*Constant*	2579,529					
*ROC curve analysis*						
*Area under the ROC curve (AUC) *	0.917					
*Standard Error*	0.00479					
*95% Confidence interval*	0.908 to 0.926					

**Fig 9:**
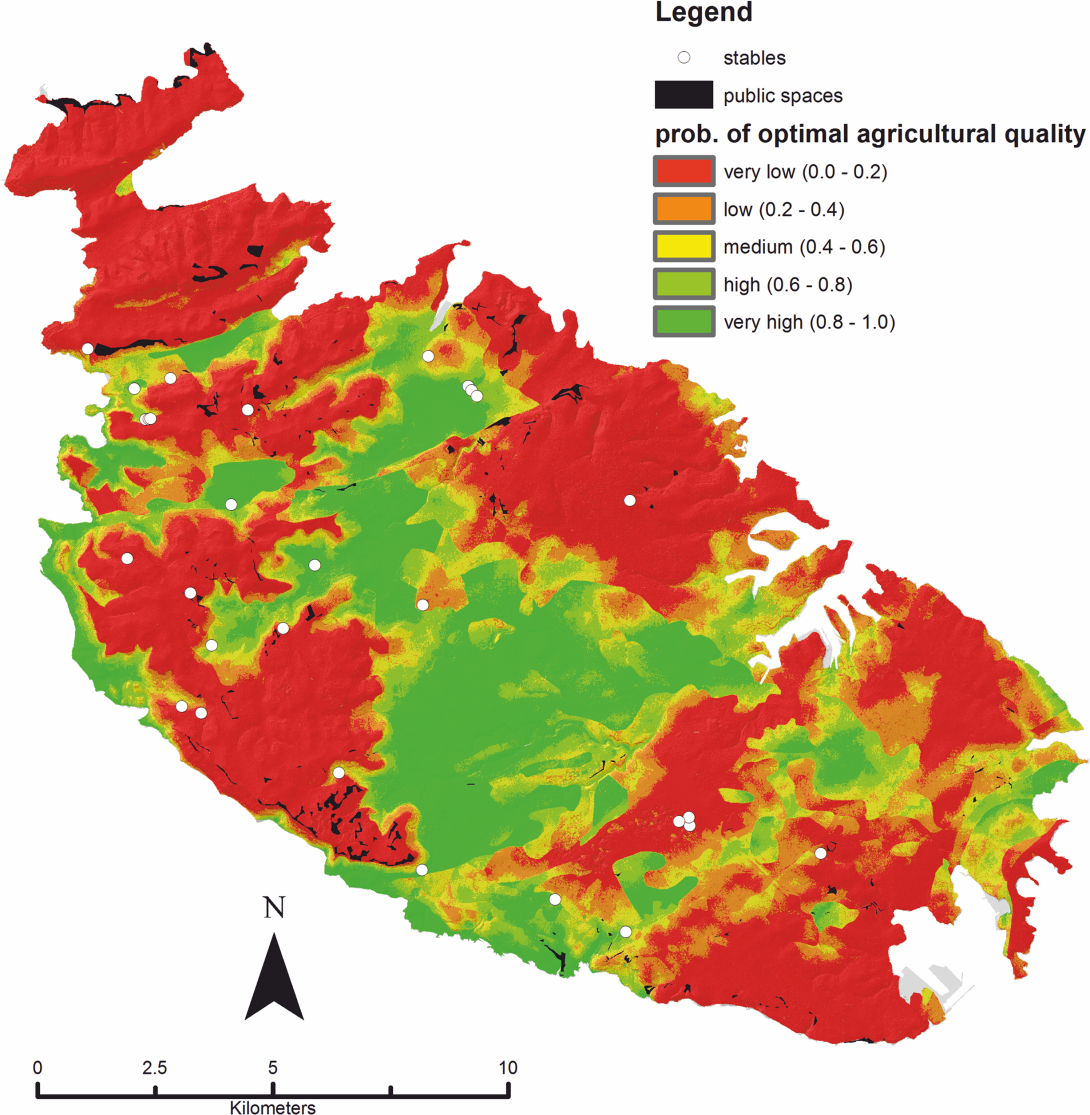
Fitted logistic regression model. Fitted cabreo model; colours represent the probability (ranging between 0.0 and 1.0) for optimal agricultural quality. Probability values have been categorized into five classes spanning from very low to very high. The location and extent of public spaces in mid-1800s Malta, and the location of farmhouses in which stables are recorded, is also shown. The latter information derives from the sample of cabreo maps described in the text. Image created by the authors in ESRI’s ArcGIS 10.1; no copyrighted material was used.

The estimated coefficients and the constant were fed into ArcGIS via the *Raster Calculator* facility in order to produce a raster representing the fitted model. The raster has been given a colour scale reflecting the probability for ‘optimal’ land quality, ranging from the lowest (red = 0.0 probability) to the highest (green = 1.0 probability) (Fig 9).

As for the predictors related to topography, elevation and slope have a negative effect on the chances for optimal agricultural quality: when they increase by 1 unit, the odds of optimal agricultural quality decrease by a factor of 0.96 and 0.97 respectively (or, put another way, the odds are 0.96 and 0.97 times as small for an additional metre of elevation and degree of slope, respectively). The sine of the aspect turns out to have a positive effect according to the model: as it increases (i.e., turning from west to east), the odds of optimal agricultural quality increase by 1.42. The distance to the coastline and to the nearest geological fault have an opposite effect on the outcome of the dependent variable. As the distance to the coastline increases by 1 unit, the odds of optimal agricultural quality increase by 2.42, whereas 1-unit increase in distance to the nearest fault line decreases the odds by 0.09.

The fitted model also allows to assess the effect of different soil types on the outcome of the dependent variable. Brown Rendzinas soils are associated with an increase in the odds of optimal agricultural quality by a factor of 2.31, and the same holds true for Carbonate Raw and Xerorendzinas soils, by a factor of 2.62 and 9.23 respectively. On the other hand, Terra Rossa soils are associated with a decrease in the odds by 0.46.

As for the cultural predictors related to land accessibility (i.e., distance to the road network), the distance to the nearest secondary road and to the nearest footpath was found to have a negative effect on the odds of optimal agricultural quality. As the model indicates, a 1-unit increase in those variables translates into a decrease of the odds by 0.19 and 0.41 respectively. On the other hand, a 1-unit increase in the distance to the nearest minor road is associated with an increase in the odds of optimal agricultural quality by a factor of 4.88.

## Discussion

The model made it possible to isolate a host of variables that had an influence, either positive or negative, on the suitability for agriculture at the time when the cabreo was created. The literature reviewed earlier in this work provides grounds to understand the negative impact of elevation and slope. At least three factors may account for the negative contribution of elevation. As already mentioned, relative elevation affects water retention: terrain at higher elevation drain more readily and receives less water from upslope. In addition, terrain at lower elevations is generally better sheltered against the negative effects of winds [37]. Furthermore, changes in elevation go hand-in-hand with changes in temperature. The negative impact of the slope on agricultural quality is consistent with many aspects of the reviewed literature. Steep slopes can in fact be considered a *handicap* [32] for agricultural areas not only because they hamper the use of mechanical devices [30], but also because slope influences solar radiation, water infiltration rates, surface runoff, moisture, erosion, and depth of soils. The practice of terracing aims at reducing some of these negative effects [110,111]. The modelled increase in the odds of optimal agricultural quality as the terrain starts to gently slope down is consistent with Rolé’s [38] remarks describing an increase in productivity as ones moves from mid-slope to the valley, which is also characterised by deeper soils [62]. This was already stressed by Lang [61], who noted that alluvial flats, flatter lands and (notably) *damper* valley bottoms were usually heavily cultivated in Malta. An interesting parallel is provided, for instance, by northwestern Keos (in the Aegean area) where in the early 1900s agricultural suitability was shaped by different factors, including physical ones. As Cherry et al. [68] argue, valley bottoms were preferred because they were more accessible, easier to irrigate, and characterised by deeper soils.

The influence of eastern aspects as opposed to western ones, with the former being more suitable for agriculture, turns out to be an interesting result. On the grounds of what was reviewed earlier in this study, it can be posited that east-facing slopes were more favourable for being relatively cooler than those exposed to south and west, so retaining more soil moisture. This can be considered a crucial factor for agriculture especially in a climate like that of the Maltese archipelago, where summers are hot and dry, and springs are characterised by rainfall deficit [62], with a resulting high rate of evapo-transpiration [112]. On the other hand, the decrease in the odds of optimal agricultural quality associated with west-facing slopes is consistent with the fact that slopes facing west and southwest receive a greater amount of solar radiance [43], resulting in drier conditions and in a different microclimate at ground level. Given that young plants and seedlings are killed by *heat stresses* [31], e.g. by temperature exceeding 38 degrees Celsius [37], it makes perfect sense that the cooler slopes have a positive effect on the chances for optimal agricultural quality. Eastern exposures also benefit from morning sun that allows plants to dry from dew or rain sooner than those on western slopes. Moreover, in the study area, east-facing slopes are more sheltered from the prevailing winds. Meteorological data gathered between 1997–2006 indicate that the most frequent direction is from northwest, followed in frequency by westerly winds [113].

The positive impact of the distance to the coastline can be explained in the light of what was touched upon previously. Being relatively distant from the coast implies being less prone to sea-spray and salt-laden air. On the other hand, the model shows that the distance to the nearest geological fault line has a negative impact on the odds of optimal agricultural quality. Since this predictor has been used as proxy for fresh water availability, the model seems to coherently indicate that being progressively far from a fault line translates into a decrease in the chances to have access to fresh water. This can be easily considered a crucial factor for agricultural development in an arid climate such as the Maltese one [62].

Another interesting achievement of the study is the possibility of assessing the contribution of different soil types to the agricultural quality as recorded in the cabreo. The model confirms many of the empirical observations made by Lang in the 1960s, and there seems to be a good correspondence between his remarks on land productivity and the agricultural suitability as predicted by the fitted model. The soils defined by Lang as giving satisfactory crops were Xerorendzinas, Brown Rendzinas, and Carbonate Raw [61]. The first two belong to those Rendzina soils that, elsewhere in Europe [114,115], are regarded as having favourable physical properties, characterised by high water infiltration rates when wet and high water-holding capacity, resulting in high biological activity and high natural fertility. According to the model, they are associated with an increase in the odds of optimal agricultural quality, which is consistent with Lang’s remarks. The latter are consistent with the model’s results also in relation to Terra Rossa soils, which were *rather dry*, *compact*, *and difficult to cultivate* and were usually *left uncultivated*, or used for bird catching or sheep/goat grazing [61]. Notably, the model proposed in this study pointed to this type of soil as a negative factor for optimal agricultural quality. It is worth stressing that the areas for which the model estimated a low probability of optimal agricultural quality can be nonetheless thought of as being potentially good for purposes other than agriculture. Indeed, historical and ethnographic sources from Malta and elsewhere reveal that thin-soiled and scrub-covered karstland was used for a variety of purposes that turned an apparently unproductive landscape into an important part of the agrarian economy. They actually provided grazing grounds for sheep and goats, quarried stone for construction, brushwood for fuel, apart from herbs, greens, wild game, and flowering plants for bee pasture [116–118]. It is also worthy of note that in our cabreo sample farmhouses incorporating pens for traction animals and others (*stalle*, or stables) tend to occur in areas with predicted low probability for optimal agriculture, or on the fringe of good agricultural zones (see the aforementioned Fig 9). Moreover, public spaces (*spazi pubblici*) or wasteland occur amongst such uncultivated areas. If this form of opportunistic exploitation of a common resource and resulting economic return is often hard to assess, the investment in demarcating such apparently unproductive areas with rubble walls and ensuring access to them for humans and herds through walled paths or tracks is hard to miss. As a matter of fact, our data indicate that there is a tendency for stables to be located close to public spaces. A randomized test, performed with the aid of the PASSaGE v.2 program [119], shows that the average minimum distance is 635 m, which is significantly smaller (*p*: <0.01) than the randomized average minimum distance of 1141 m, calculated across 999 permutations. It would appear, therefore, that there is a ‘symbiosis’ between the location of farmhouses incorporating stables, on the one hand, and these public spaces on the other. Indeed, we are currently investigating the latter aspect in the framework of the same project to which reference has been made earlier on in this study. Employing the fitted cabreo model as constrain, we will use GIS to isolate potential foraging routes from stables toward those areas known from ethnographic accounts for being used as pastures. Among other things, the likely routes (generated via *Least-Cost Path* analysis) will be compared to information derived from interviews of local shepherds and to evidence regarding the spatial distribution of disappeared villages originally connected to the movement of flocks across the landscape [120]. All in all, our research into the pastoral foraging landscape will aim at exploring the ways in which zones flagged by the cabreo model as *non optimal* for agriculture could have been used for other aspects of the economic exploitation of the Maltese landscape.

The model’s results regarding land accessibility are also interesting. The analysis formally shows that accessibility had an effect on agricultural suitability; in particular, the more distant a plot was from secondary roads, the lesser the odds of optimal agricultural quality. This makes sense inasmuch the secondary road network can be thought of as allowing a gradual shift from urbanized areas to more peripheral zones, going deeper into the landscape relative to the main road system (which, remarkably, turned out not to have a significant contribution to the model), and providing access to the countryside. Seen from this perspective, it is not by chance if being distant from secondary roads (i.e., being in less accessible plots of land) decreases the odds of optimal agricultural quality, according to the model. While this proves an interesting new acquisition for the study area, it is something that has been stressed in ethnoarchaeological studies of rural settlements and land use elsewhere in the Mediterranean. For instance, Cherry et al. [68] have stressed that in northwestern Keos land accessibility was one of the factors influencing the decision to cultivate a particular land as of early 1900s, before the use of motorized transport became widespread. This situation now finds an interesting parallel in mid-1800s Malta.

The issue of land accessibility and its interpretation holds true for the distance to the nearest footpath, which has a similar negative effect: as seen, the increase in distance to footpaths is associated with a decrease in the odds for optimal land quality. It must be noted that the model’s results for the distance to the nearest minor road seem counterintuitive, and deserve comments. If seen from the standpoint of the secondary road network and of its ability to make the landscape accessible, one would expect minor roads to have an effect similar to that of secondary roads. Yet, unlike secondary roads, the distance to the nearest minor road is associated with an increase in odds, i.e. the larger the distance the larger the odds for optimal quality. A review of the literature shows that proximity to roads may alter the chemical composition of adjoining soils, leading to a decrease in fertility [121–123]. Besides chemical factors, physical ones may also account for a decrease in agricultural suitability: S. Vella [62], for instance, argues that roads increase the quantity and velocity of runoff, enhancing soil erosion and damage to rubble walls. She goes on saying that loose materials used for surfaced tracks release gravel, which may increase the erosive ability of runoff. From this standpoint, the model result as to distance to the nearest minor road may prove less counterintuitive, and may help explaining the modelled increase in the odds of optimal agricultural quality as one moves away from minor roads. Such a scenario may explain what is happening in the immediate vicinity of a road. However, it may well be that minor roads occur in areas of garrigue and karstland which in the model correspond to an area less favourable for agriculture. Optimal land quality would be found further away from these, downslope or in valley bottoms, areas highlighted by the model for their agricultural suitability.

All in all, our findings shed considerable new light on some of the questions posed in the introduction to this paper. One of the striking characteristics displayed both by the archival evidence and by the resulting model is the wide variability in land quality that is evident even over small distances. The rugged profile of the island presents dramatically different micro-environments which typically range from exposed plateaux, to well-watered but steep clay slopes, to more sheltered valley bottoms with rich and deep alluvial and colluvial fills. Each of these micro-environments may present very different agricultural affordances. As stressed, some may be unsuitable for crop cultivation, but ideal for grazing for sheep and goat. The fragmented and variable nature of the Maltese landscape is interestingly an enduring characteristic, which would have been no less variable in more remote periods, and even during prehistory. Each of these environments may of course have undergone considerable transformations over long periods of time, resulting in very different agricultural affordances in different periods. However this does not alter the basic fact that the Maltese landscape, like many small island environments, presents a range of different agricultural opportunities in close juxtaposition. Mixed subsistence strategies, such as those combining animal grazing and food collecting on more inhospitable areas, with crop cultivation in more sheltered and favourable zones, appear to be better suited to such an environment. This characteristic should be taken into account in any research efforts to reconstruct prehistoric and ancient subsistence strategies in such landscapes.

## Conclusions

This work has sought to use for the first time mid-1800s cabreo maps as a basis to develop a statistical model in a GIS environment to understand possible determinants of agricultural quality in Malta before heavy mechanization. Logistic regression modelling has made it possible to gauge the effect of a host of topographic and cultural factors on the agricultural quality, enabling us to build a predictive model for the entire study area. It has been therefore possible to isolate a negative contribution of some topographic factors such as elevation, slope, distance to the nearest geological fault line (used as proxy for fresh water availability). Other factors, such as distance to the coast, turned out to have a positive effect on the chances for optimal agricultural quality. The model has also indicated that different soil types had different effects on agricultural quality, with Terra Rossa proving to have a negative influence on agriculture relative to other types of soils. This proved to be consistent with observations published almost a century later, hence pointing to an interesting continuity in some constrains to optimal agriculture in the Maltese landscape. Remarkably, the analysis also showed that cultural factors, such as roads network and related mobility and landscape accessibility, had a constraining role as well, in a way consistent with findings in other Mediterranean islands. It has also been stressed that wide zones flagged as non-optimal for agriculture by the model must have been nonetheless good for a host of other activities such as grazing grounds, quarrying, fuel procurement, as both documentary evidence and the location of farmhouses incorporating stables indeed indicate. They were therefore part of a broader economic picture. All in all, our model has shown that different factors are likely to have shaped the agricultural landscape of the Maltese Islands. A host of topographic and cultural factors, the latter related to human mobility and landscape accessibility, did contribute to differential agricultural suitability. They provided the bases to the creation of that fragmented and extremely variegated agricultural landscape that is the hallmark of the Maltese Islands.

## Supporting information

S1 Text Dataset. (TXT)

S2 Text Predictors selection and model validation. (DOC)
