# Supplementary material for: The use of geographic information system and 1860s cadastral data to model agricultural suitability before heavy mechanization. A case study from Malta
Source: PLoS One. 2018 Feb 7;13(2):e0192039. doi: 10.1371/journal.pone.0192039 (PMC5802886; doi:10.1371/journal.pone.0192039)
Supplement: S2 Text — (DOC) [file pone.0192039.s002.doc]

**S2 Predictors selection and model validation**

**Predictors selection and model validation**

The Rizopoulos’s *boot.StepAIC* R package [1] package implements the model selection devised by Austin and Tu [2], widely used in literature [3–6]. The procedure draws *n* bootstrap samples from the original dataset. For the purposes of the present study, *n* was set at 1000. The model is fitted on each bootstrap dataset, each time using a backward stepwise procedure that uses the Akaike’s Information Criterion (a goodness-of-fit measure, corrected for model complexity) to select the most parsimonious model. In other words, for each bootstrap sample, all predictors are initially entered in the model and then they are iteratively removed to assess if the AIC improves (i.e., decreases) when each predictor is taken out from the model. If a variable is removed, the contribution of the remaining predictors is reassessed, and the procedure continues until no further AIC improvement takes place [7]. The proportion of bootstrap samples in which each predictor was identified as an independent predictor is determined, and then predictors are ranked on the basis of that proportion. If variables are truly independent predictors, they should be identified as predictors in the majority of the samples. Furthermore, the procedure keeps track of the proportion of times in which each predictor has a negative or positive coefficient across all the bootstrap samples. This allows the stability of the estimated coefficient to be assessed, since the expectation for variables being truly independent predictors is that their coefficients are either all positive or all negative [2]. All in all, the described method allows assessment of the *posterior probability of each variable being included in the model* [2].

The method described by Arboretti Giancristofaro-Salmaso [8], which has been implemented in *R* [9,10], consists of the following steps: 1) the whole dataset is split into two random parts, a fitting (75%) and a validation (25%) portion; 2) the model is fitted on the fitting portion (i.e., its coefficients are computed considering only the observations in that portion) and its performance is evaluated on both the fitting and the validation portion, using AUC as performance measure ; 3) steps 1-2 are repeated *n* times (1000 in this study), eventually getting a fitting and validation distribution of the AUC values. The former provides an estimate of the performance of the model in the population of all the theoretical training samples; the latter represents an estimate of the model’s performance on new and independent data.

**References**

1. Rizopoulos D. Package ‘bootStepAIC’ [Internet]. 2009. Available: https://cran.r-project.org/package=bootStepAIC

2. Austin PC, Tu J V. Statistical Bootstrap Methods Practice for Developing Predictive Models. Am Stat. 2004;58: 131–137.

3. Luder MT, Pittet I, Berchtold A, Akré C, Michaud PA, Surís JC. Associations between online pornography and sexual behavior among adolescents: Myth or reality? Arch Sex Behav. 2011;40: 1027–1035. doi:10.1007/s10508-010-9714-0

4. Pittet I, Berchtold a, Akré C, Michaud P, Surís J-C. Are adolescents with chronic conditions particularly at risk for bullying? Arch Dis Child. 2010;95: 711–716. doi:10.1136/adc.2008.146571

5. Cooke CR, Shah C V, Gallop R, Bellamy S, Ancukiewicz M, Eisner MD, et al. A simple clinical predictive index for objective estimates of mortality in acute lung injury. Crit Care Med. 2009;37: 1913–1920. doi:10.1097/CCM.0b013e3181a009b4

6. Dunn DC, Stewart K, Bjorkland RH, Haughton M, Singh-Renton S, Lewison R, et al. A regional analysis of coastal and domestic fishing effort in the wider Caribbean. Fish Res. 2010;102: 60–68. doi:10.1016/j.fishres.2009.10.010

7. Field A, Miles J, Field Z. Discovering Statistics Using R. Thousand Oaks: Sage; 2012.

8. Arboretti Giancristofaro R, Salmaso L. Model performance analysis and model validation in logistic regression. Statistica. 2003;63: 375–396. Available: http://rivista-statistica.unibo.it/index.php/rivista-statistica/article/view/358

9. R Team Core. R: A language and environment for statistical computing. [Internet]. Vienna: R Foundation for Statistical Computing; 2013. Available: https://www.r-project.org/

10. Alberti G. ‘model.valid’: R function for cross-validated AUC. 2016. doi:10.13140/RG.2.1.4636.6480
